# Brazilian Portuguese Language version of the “Tinnitus Handicap Inventory”: Validity and Reproducibility

**DOI:** 10.1016/S1808-8694(15)31048-X

**Published:** 2015-10-19

**Authors:** Letícia Petersen Schmidt, Vanessa Niemiec Teixeira, Celso Dall’Igna, Daniel Dallagnol, Mariana Magnus Smith

**Affiliations:** 1 MD. MsC student of the Post-graduation program in Surgery of the Medical School of the Federal University of Rio Grande do Sul. Fellow in Otology from the Department of Otorhinolaryngology of the Porto Alegre University Hospital.; 2 MD. Otorhinolaryngology Resident at the Porto Alegre University Hospital.; 3 PhD in Medicine. Associate Professor of Otorhinolaryngology - Medical School of the Federal University of Rio Grande do Sul. Head of the Otorhinolaryngology Department of the Porto Alegre University Hospital.; 4 Medical Student - Rio Grande do Sul Federal University - Medical School.; 5 MD. MsC in the Medical Sciences Post-graduation program of the Federal University of Rio Grande do Sul. Fellow in Laryngology of the Otorhinolaryngology Department - Porto Alegre University Hospital. Department of Otorhinolaryngology - Porto Alegre University Hospital - Federal University of Rio Grande do Sul.

**Keywords:** thi, portuguese version, tinnitus

## Abstract

Tinnitus can greatly impact an individual’s life quality and it is very difficult quantify. **Aim:** To determine the reproducibility and validity of a Brazilian Portuguese version of the *Tinnitus Handicap Inventory* (THI), a self-applicable questionnaire which assesses tinnitus impact on patients’ life quality. **Materials and Methods:** This was a prospective transversal study. The questionnaire was translated into Portuguese and cross-culturally adapted to the Brazilian environment according to internationally recommended methods. The Portuguese version of the THI was answered by 180 patients who complained of tinnitus. Reproducibility was assessed using the Cronbach’s Alpha Calculation; and the validity was assessed by means of the Beck Depression Inventory (BDI), calculating the Pearson correlation coefficient. **Results:** The Portuguese version of the THI showed high internal validity, comparable to the original version. A high correlation was observed between the THI and the BDI. **Conclusion:** The Brazilian Portuguese version of THI is a valid and reproducible tool used to quantify how tinnitus impact the life quality of those Brazilian patients who complain of this symptom.

## INTRODUCTION

The Tinnitus Handicap Inventory (THI) is a questionnaire developed by Newman et al.^1^ in 1996, compounding 27 questions, with a score that varies between 0 and 100, the higher the score, the greater is the tinnitus impact on the individual’s life quality. It is a fast, easy to apply and easy to interpret type of measure. It has been broadly used in the clinical setting to assess patients with tinnitus, in order to quantify the trouble related to such symptom and to assess treatment response. As with most questionnaires, this one was also created in English, to be used with the population that speaks the language. Therefore, in order to use it in our country, we must follow rules that have been previously established in the literature regarding its translation; and later, its measuring properties must be shown within a specific cultural context, as it was the case when it was validated in Spanish and Danish^2^, ^3^.

The choice of such tool is based on the need to have such a well designed questionnaire translated into Portuguese, is capable of quickly and definitively assessing the trouble tinnitus causes on the lives of those people who have it, of which validity and reproducibility have already been proven.

This paper aims at determining the reproducibility and validity of the THI Portuguese version.

## MATERIALS AND METHODS

We assessed 180 patients complaining of tinnitus with or without hearing loss, being followed at the Department of Otorhinolaryngology of the Porto Alegre University Hospital (HCPA), between 2001 and 2004. The sample was made up of individuals from both genders, 64% were women. Age average was 55.85 years, varying between 19 and 77 years. Only 10% of our sample presented hearing thresholds within normal range.

Craig Newman, author of the original questionnaire in English previously authorized us to validate it into Portuguese.

**Figure 1:**
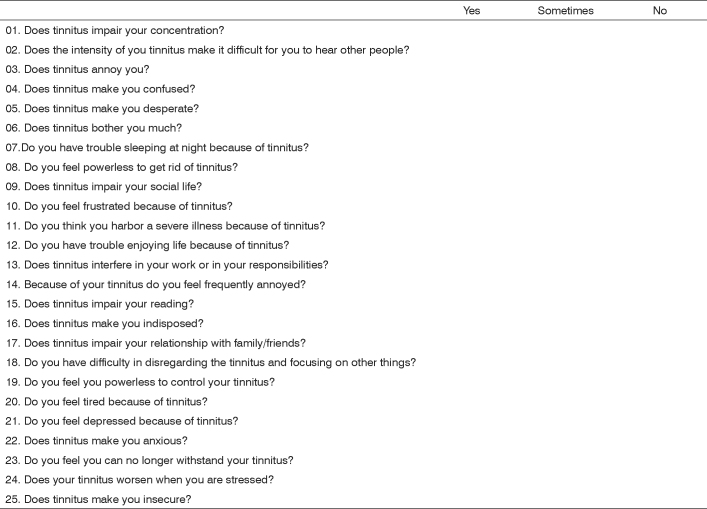
THI Portuguese language version.

We then developed a translation protocol based on papers published in the literature which discuss translation methods for questionnaires into other languages, stressing the conceptual translation, and not strictly to the letter (Figure 1).

This project was approved by the Ethics Committee of the HCPA (protocol # 06-027).

We assessed the percentages of “yes”, “sometimes” and “no” answers and we then calculated the reproducibility using the Cronbach Alpha Method.

We used the THI score relation with a broadly used scale: the Beck scale (Beck depression inventory)4 by means of the Pearson’s coefficient.

## RESULTS

**Figure 2:**
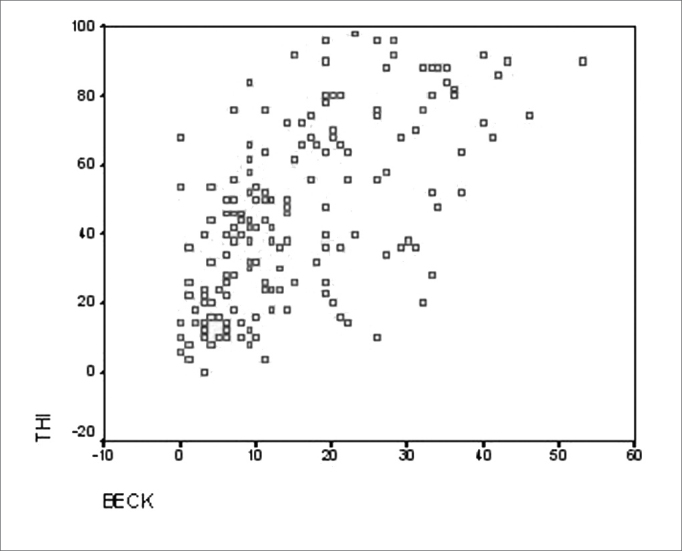
Chart showing the correlation between THI and the Beck scale.

The Cronbach Alpha coefficient was 0.929, with a total items correlation varying between r= 0.924 and r= 0.931, showing its reproducibility. The correlation coefficient between total THI and the Beck scale was r= 0.68 (p<0.01), confirming its validity (Figure 2).

## DISCUSSION

Understanding tinnitus and its impact on the life quality of patients is of the uttermost importance. So far, the tool used for its assessment was found only in English, Spanish and Danish. Today, it is widely agreed that a careful translation is not enough in order to validate an assessment tool, since the terms to be used should be adapted for the social and cultural settings of the population it will serve. It is also necessary that its psychometric measures be tested within a specific cultural context, since each society has its own beliefs, behaviors, and social practices that guide their behaviors and attitudes, thus reflecting a country’s culture. When the translation of a questionnaire is proposed, it must be simple and clear, without loosing its equivalence towards cultural issues. This is what we did with this translation. The present study proved the internal coherence of the Portuguese version.

The THI Portuguese translation and its adaptation to the socio-economical and cultural settings of our population, as well as the proof of its reproducibility and validity make this tool an additional and useful parameter that may be used not only for the initial assessment of tinnitus patients, but also to control their responses to different treatment modalities.

## CONCLUSION

The THI Portuguese version is a valid and reproducible tool to be used in assessing Brazilian patients who came to use with tinnitus. It allows us to quantify how much this symptom impacts the life quality of these patients.
